# Regulator of G Protein Signalling 4 (RGS4) as a Novel Target for the Treatment of Sensorineural Hearing Loss

**DOI:** 10.3390/ijms22010003

**Published:** 2020-12-22

**Authors:** Christine Fok, Milan Bogosanovic, Madhavi Pandya, Ravindra Telang, Peter R. Thorne, Srdjan M. Vlajkovic

**Affiliations:** Department of Physiology and The Eisdell Moore Centre, Faculty of Medical and Health Sciences, The University of Auckland, Private Bag 92019, Auckland 1142, New Zealand; c.fok@auckland.ac.nz (C.F.); milan.bogosanovic@uq.net.au (M.B.); m.pandya@auckland.ac.nz (M.P.); r.telang@auckland.ac.nz (R.T.); pr.thorne@auckland.ac.nz (P.R.T.)

**Keywords:** sensorineural hearing loss, noise-induced cochlear injury, cochlear rescue, otoprotection, adenosine A_1_ receptor, regulator of G protein signalling 4, CCG-4986, intratympanic drug delivery

## Abstract

We and others have previously identified signalling pathways associated with the adenosine A_1_ receptor (A_1_R) as important regulators of cellular responses to injury in the cochlea. We have shown that the “post-exposure” treatment with adenosine A_1_R agonists confers partial protection against acoustic trauma and other forms of sensorineural hearing loss (SNHL). The aim of this study was to determine if increasing A_1_R responsiveness to endogenous adenosine would have the same otoprotective effect. This was achieved by pharmacological targeting of the Regulator of G protein Signalling 4 (RGS4). RGS proteins inhibit signal transduction pathways initiated by G protein-coupled receptors (GPCR) by enhancing GPCR deactivation and receptor desensitisation. A molecular complex between RGS4 and neurabin, an intracellular scaffolding protein expressed in neural and cochlear tissues, is the key negative regulator of A_1_R activity in the brain. In this study, Wistar rats (6–8 weeks) were exposed to traumatic noise (110 dBSPL, 8–16 kHz) for 2 h and a small molecule RGS4 inhibitor CCG-4986 was delivered intratympanically in a Poloxamer-407 gel formulation for sustained drug release 24 or 48 h after noise exposure. Intratympanic administration of CCG-4986 48 h after noise exposure attenuated noise-induced permanent auditory threshold shifts by up to 19 dB, whilst the earlier drug administration (24 h) led to even better preservation of auditory thresholds (up to 32 dB). Significant improvement of auditory thresholds and suprathreshold responses was linked to improved survival of sensorineural tissues and afferent synapses in the cochlea. Our studies thus demonstrate that intratympanic administration of CCG-4986 can rescue cochlear injury and hearing loss induced by acoustic overexposure. This research represents a novel paradigm for the treatment of various forms of SNHL based on regulation of GPCR.

## 1. Introduction

Hearing loss is the most prevalent form of sensory impairment, affecting about 466 million people worldwide including 34 million children [1]. Most of the hearing loss is sensorineural due to disease, degeneration, or trauma to the cochlea of the inner ear [2]. Treatment options for sensorineural hearing loss (SNHL) are currently limited to prosthetic devices such as hearing aids and cochlear implants. Both devices can partly restore auditory function, but these have limitations because the ear remains damaged. There is thus a significant need for the development of effective therapies to prevent cochlear damage and hearing loss or restore cochlear sensorineural structure and hearing. We have identified that signalling pathways activated by adenosine receptors are important regulators of cellular responses to injury in cochlear tissues. Animal studies reveal that stimulation of the A_1_ adenosine receptor (A_1_R) is particularly promising for the treatment of acute noise-induced cochlear injury [3,4] and other forms of SNHL such as from cytotoxic drugs, including cisplatin and aminoglycoside antibiotics [5,6]. The principal advantage of this approach is that the A_1_R stimulation affects multiple mechanisms of cochlear injury (e.g., oxidative stress, glutamate excitotoxicity, activation of apoptotic pathways), thus providing comprehensive protection from SNHL [7]. One of the issues with advancing these approaches to clinical applicability is the delivery of receptor agonists to the inner ear and their suitability for long-term therapy.

As an alternative to the use of exogenous A_1_R agonists, we have previously considered other strategies that would regulate the action of endogenous adenosine, for example by manipulating intracellular adenosine metabolism [8]. Recently, we have identified a novel otoprotective paradigm based on increasing A_1_R responsiveness to endogenous adenosine, which can be achieved by inhibiting the Regulator of G protein Signalling 4 (RGS4). RGS is a large family of proteins that inhibit signal transduction pathways initiated by G protein-coupled receptors (GPCR) including A_1_R [9,10,11]. RGS increase the intrinsic GTPase activity of G proteins and thus enhance G protein inactivation and promote receptor desensitisation [9].

In the past two decades, RGS proteins have received increasing interest as potential drug targets in cardiovascular disease, CNS disorders and several types of cancer [12,13,14,15]. Targeted inhibition of RGS proteins could potentially provide a way to fine-tune GPCR signalling by potentiating or prolonging the effect of receptor agonists. This approach could also be used to enhance endogenous ligand effects on GPCR. A few small molecule RGS inhibitors have been identified, particularly in the well-studied RGS4 family [16,17]. The selectivity of RGS proteins is mediated either through direct interaction with target receptors, or through selective interactions with accessory proteins [16]. For example, the neurabin-RGS4 molecular complex regulates A_1_R signalling events in the brain. Neurabin is an intracellular scaffolding protein (protein phosphatase 1 regulatory inhibitor subunit 9a, PPP1R9A) expressed in neural tissues which facilitates interactions of RGS4 with the A_1_R [18]. After A_1_R stimulation by endogenous or exogenous ligands, neurabin forms a complex with RGS4 and recruits it to the cell surface to the A_1_R [18]. Disruption of the neurabin-RGS4 complex, either by genetic deletion of neurabin or by selective inhibitors of RGS4, enhances A_1_R signalling even without administration of exogenous A_1_R ligands. Mice with genetic deletion of neurabin are protected against kainate-induced seizures, evidenced by reduced severity and occurrence of seizures, improved neuronal survival and overall lower mortality [18]. Similarly, this anticonvulsant and neuroprotective effect is also conferred by CCG-4986, a small molecule inhibitor of RGS4 [18]. The neurabin/RGS4 complex thus appears to be the key regulator of A_1_R activity in the brain and a promising neuroprotective target.

Here, we investigated an otoprotective strategy based on inhibition of the neurabin/RGS4 complex in the cochlea, with the aim to increase A_1_R responsiveness to endogenous adenosine released from cochlear tissues during acoustic stress. This novel otoprotective strategy is based on intratympanic injection of a small molecule RGS4 inhibitor to the round window membrane (RWM) of the cochlea.

## 2. Results

### 2.1. Expression and Immunolocalisation of RGS4 and Neurabin I in the Cochlea

**Figure 1:**
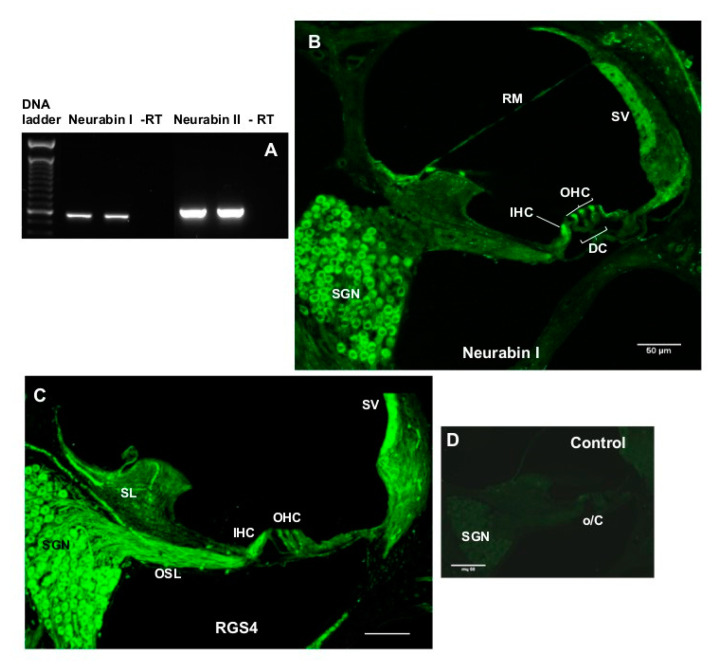
(**A**) Expression of Neurabin I and II isoforms in the rat cochlea. PCR products (shown in duplicates) correspond to the neurabin I (520 bp) and neurabin II (561 bp) isoforms, respectively. In the absence of reverse transcriptase (-RT controls) no amplification was observed. (**B**) The most prominent neurabin I immunostaining was observed in sensory inner hair cells (IHC) and outer hair cells (OHC) in the organ of Corti (o/C) and spiral ganglion neurons (SGN). (**C**) The RGS4 antibody also demonstrated predilection for sensory hair cells (IHC and OHC) and SGN. RGS4 immunofluorescence was also observed in the auditory nerve fibres in the osseous spiral lamina (OSL) and blood vessels in the spiral limbus (SL). (**D**) Control section where the primary antibody was replaced by IgG isotype control. Abbreviations: DC, Deiters’ cells; RM, Reissner’s membrane; SV, stria vascularis. Scale bars, 50 μM.

RT-PCR demonstrated the expression of neurabin isoforms I and II in the rat cochlea (Figure 1A). Generated PCR products corresponded to the predicted sizes of DNA fragments (Table 1). Omitting reverse transcriptase (-RT) in control reactions resulted in the absence of reaction products (Figure 1A). As only the neurabin I isoform makes complexes with RGS4 and the A_1_R, immunolocalisation studies were only performed for this isoform. Neurabin I was immunolocalised in the inner and outer hair cells in the organ of Corti and cell bodies of the spiral ganglion neurons (Figure 1B). Neurabin I distribution in sensory hair cells and spiral ganglion neurons coincided with the RGS4 immunofluorescence pattern (Figure 1C). In addition, RGS4 immunofluorescence was observed in the auditory nerve fibres in the osseous spiral lamina and blood vessels in the spiral limbus (Figure 1C). No immunofluorescence was detected when the primary antibody was replaced with control mouse IgG (Figure 1D) or control rabbit IgG (not shown).

### 2.2. Auditory Brainstem Responses and the Effect of Treatment 48 Hours after Noise Exposure

Auditory brainstem responses (ABR) were used to measure auditory thresholds prior to noise exposure (baseline), and 16 days after noise exposure (final) to determine noise-induced threshold shifts.

**Figure 2:**
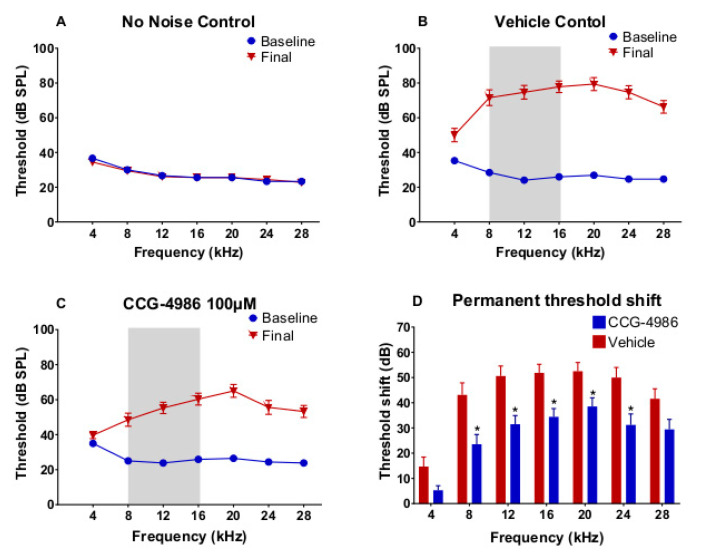
Baseline and final auditory brainstem responses (ABR) thresholds for 4–28 kHz tone pips in animals exposed to octave band noise (8–16 kHz, 110 dB SPL) for 2 h and non-exposed animals. Grey area denotes noise band. (**A**) Controls exposed to ambient noise (55–65 dB SPL) in the animal facility. (**B**) Noise-exposed vehicle-treated control group. (**C**) Animals treated with CCG-4986 (100 μM) 48 h post-exposure. (**D**) Comparison of permanent threshold shifts 16 days post-exposure in drug- and vehicle-treated animals. Data presented as mean ± SEM. No noise control, *n* = 10; Vehicle control, *n* = 16; CCG-4986, *n* = 17. * *p* < 0.05; Two-way ANOVA followed by Holm–Sidak post-hoc test.

The baseline auditory thresholds were similar in noise-exposed and non-exposed animals (Figure 2A–C). The control group exposed to ambient noise levels in the animal facility showed no change in ABR thresholds 16 days after initial ABR measurement (Figure 2A). In contrast, exposure to octave band noise (8–16 kHz at 110 dB SPL) for two hours induced a permanent threshold shift (PTS) in both drug- and vehicle-treated animals (Figure 2B–D). In the control vehicle-treated group, the average threshold shift was between 40 and 50 dB at frequencies above 4 kHz (Figure 2B,D). ABR thresholds were still elevated after treatment with CCG-4986 (100 μM), but to a lesser degree than in control drug vehicle-treated animals (Figure 2C). At mid-to-high frequencies (8–28 kHz), CCG-4986 treatment reduced noise-induced PTS by 10–19 dB compared to the vehicle-treated animals (Figure 2D). The greatest improvement of ABR thresholds was observed at 8–16 kHz (Figure 2D), with average threshold shift reductions of 19 dB at 8 kHz (*p* = 0.029), 19 dB at 12 kHz (*p* = 0.018) and 17 dB at 16 kHz (*p* = 0.013).

### 2.3. Input–Output Functions

**Figure 3:**
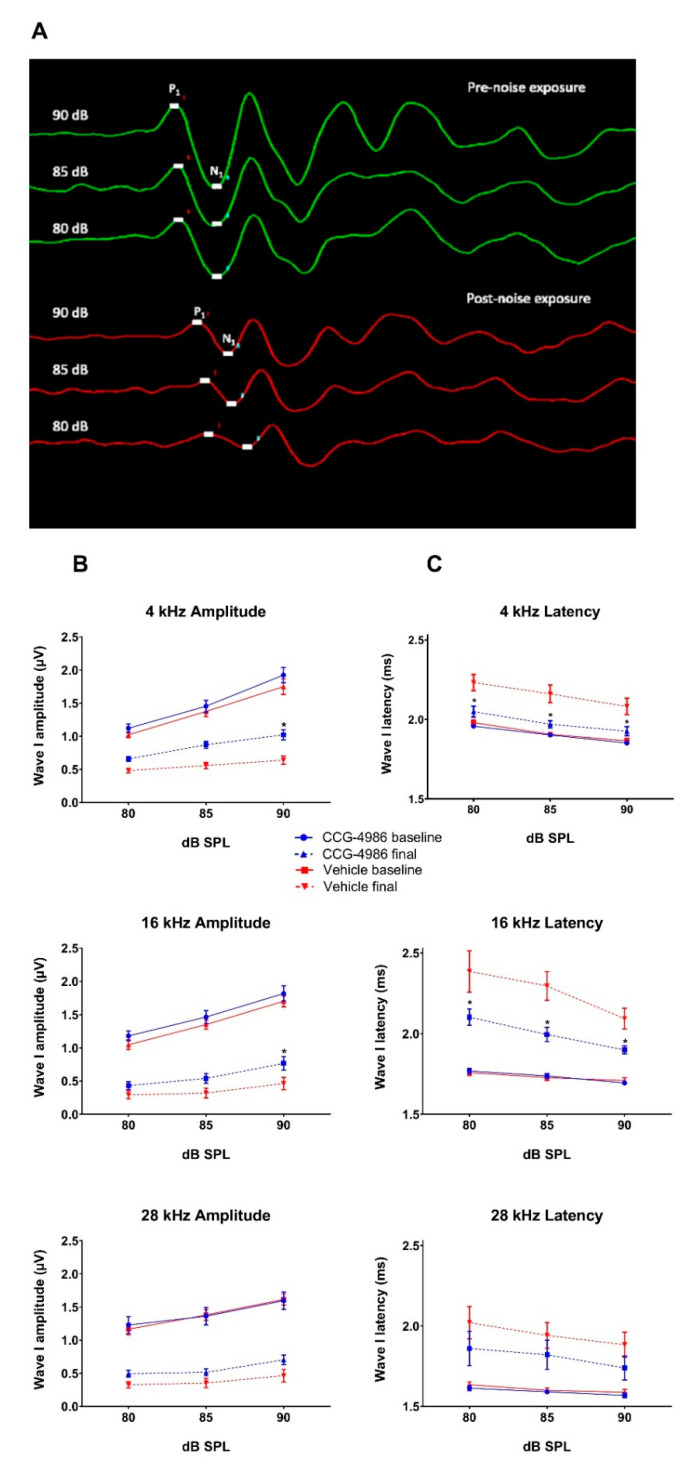
(**A**) Representative traces of ABR Wave I prior to noise exposure (green) and post-exposure (red). P_1_ represents a positive peak and N_1_ a negative peak. ABR responses were recorded at 16 kHz at suprathreshold intensities (80–90 dB SPL). Average baseline and final ABR wave I amplitudes (**B**) and latencies (**C**) at suprathreshold intensities in noise-exposed animals treated with CCG-4986 (100 μM) or drug vehicle solution 48 h after noise exposure. Solid lines represent baseline amplitudes/latencies and dashed lines represent final amplitudes/latencies. Data presented as mean ± SEM, Vehicle control, *n* = 16; CCG-4986, *n* = 17. * *p* < 0.05 Multivariate ANOVA followed by planned contrast comparisons.

To further investigate the otoprotective effects of CCG-4986 treatment, three frequencies (4 kHz, 16 kHz and 28 kHz), were selected as representative of the low, mid, and high frequency regions of the cochlea, respectively. The amplitudes and latencies of wave I were analysed at suprathreshold intensities (80, 85, and 90 dB; Figure 3A).

Prior to noise exposure, ABR Wave I amplitudes and latencies were similar in drug- and vehicle-treated rats at each test frequency. As expected, wave I amplitudes decreased, and latencies increased after noise exposure in all animals (Figure 3B,C).

The vehicle-treated control animals showed an average 60–70% reduction in wave I amplitude at 90 dB, with 16 kHz as the most affected frequency (70% reduction). CCG-4986 treatment improved wave I amplitudes compared to vehicle-treated rats, which was significant for 4 kHz (*p* = 0.03) and 16 kHz (*p* = 0.05) at 90 dB (Figure 3B). At 28 kHz the effect of CCG-4986 treatment was not significant (*p* = 0.052).

After noise exposure, wave I latency increased by 10–22% in vehicle-treated animals, with the greatest increase at 16 kHz (Figure 3C). CCG-4986 treatment significantly (*p* < 0.05) reduced wave I latencies 4 kHz and 16 kHz compared to vehicle-treated animals, although the latencies remained elevated relative to pre-noise levels (Figure 3C).

### 2.4. Hair Cell Survival

**Figure 4:**
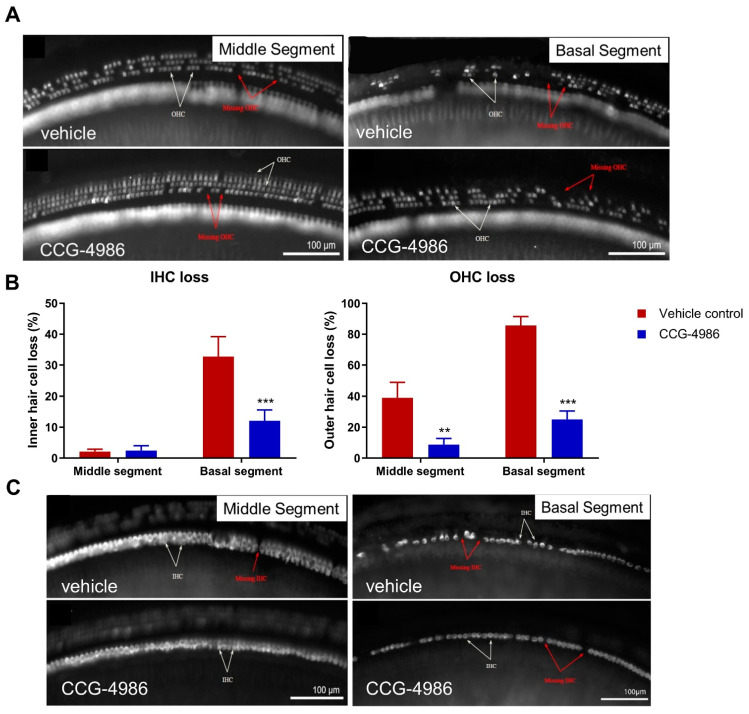
(**A**) Representative images of OHC in the middle and the basal segment of the cochlea in noise-exposed vehicle-treated and CCG-4986 treated animals. (**B**) Percentage of hair cell loss (OHC and IHC) in the middle and basal cochlear segments of vehicle- and CCG-4986 treated animals. Data presented as mean ± SEM, vehicle control *n* = 16, CCG-4986 *n* = 17. ** *p* <0.01, *** *p* < 0.0001; Two-way ANOVA followed by Holm–Sidak post-hoc test. (**C**) Representative images of IHC in the middle and the basal segment of the cochlea of vehicle-treated and CCG-4986 treated animals. White arrows point at the sensory hair cells and red arrows at spaces with missing hair cells.

As expected from exposure to the octave band noise (8–16 kHz), turn-related differences in hair cell loss were observed in both vehicle- and drug-treated animals. The loss of outer hair cells (OHC; Figure 4A) exceeded the loss of the inner hair cells (IHC; Figure 4C) in both the middle and the basal segment of the cochlea (Figure 4B). The most heavily affected region was the basal segment of the cochlea (Figure 4B), whilst there was virtually no hair cell loss in the apical segment (data not shown).

The average OHC loss in vehicle-treated animals was 38% ± 10.1% and 85% ± 5.7% in the middle and basal segments, respectively (Figure 4B). Treatment with CCG-4986 reduced OHC loss to 8.7% ± 4% (*p* = 0.0027) in the middle segment and to 25% ± 5.4% (*p* < 0.001) in the basal segment (Figure 4B).

The average IHC loss in the middle turn was low (~2%), and there was no difference between the drug- and vehicle-treated animals (Figure 4B). In contrast, the survival of IHC in the basal turn was significantly improved with CCG-4986 treatment (12.0% ± 3.5%) compared to vehicle-treated controls (32% ± 6.4%; *p* = 0.0008).

### 2.5. Synaptic Ribbon Counts

**Figure 5:**
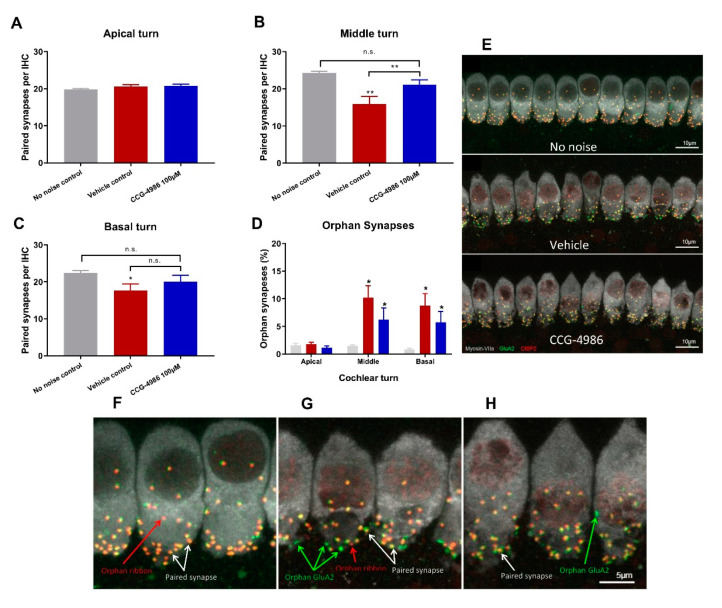
The average number of synapses per IHC at various frequency regions of the cochlea. (**A**) Number of paired synapses in the apical turn (low frequency region), (**B**) Middle turn (mid frequency region) and (**C**) Basal turn (high frequency region). (**D**) Number of orphaned synapses in all turns expressed as a percentage of total synapses per IHC. Data presented as mean ± SEM. No-noise control, *n* = 10, vehicle-treated, *n* = 16, CCG-4986 treated, *n* = 17. * *p* < 0.05, ** *p* < 0.01, n.s. not significant. One-way ANOVA followed by Holm–Sidak post-hoc test. (**E**) Representative images of IHC-auditory nerve synapses in the middle turn for controls exposed to traumatic or ambient noise. Labelling shows IHCs (grey), post-synaptic glutamate receptors (green) and pre-synaptic ribbons (red). (**F**–**H**) High power projection of IHC synapses in (**F**) No-noise controls, (**G**) Noise-exposed vehicle-treated and (**H**) Noise-exposed CCG-4986-treated animals. White arrows indicate paired ribbon synapses, green arrows orphaned post-synaptic glutamate receptors, and red arrows orphaned pre-synaptic ribbons.

Confocal imaging of afferent synapses in controls exposed to ambient noise showed turn-related differences in the average number of paired synapses per IHC (Figure 5A–C). The greatest number of synapses was found in the middle turn (Figure 5B), with an average of 24.3 ± 0.4 paired synapses per IHC. In comparison, apical and basal turns had an average of 19.8 ± 0.2 and 22.4 ± 0.6 synapses per IHC respectively (Figure 5A,C). To identify and quantify afferent synapses, whole mounts of the organ of Corti were immunolabelled with antibodies to CtBP2 (component of the presynaptic ribbon), GluA2 subunit (postsynaptic glutamate receptor) and myosin VIIa (IHC) (Figure 5E–H). 

Under ambient noise levels (no-noise controls), vast majority of synaptic ribbons were paired with the post-synaptic GluA2 receptor (Figure 5F). Only about 1% of synapses were characterised as orphan synapses (unpaired pre-synaptic ribbon or post-synaptic glutamate receptor). The percentage of orphan synapses, however increases with noise exposure (Figure 5D,E,G,H).

The number of synapses in the apical turn was similar in non-exposed and noise-exposed animals, regardless of the treatment (Figure 5A). At higher frequencies, noise exposure significantly reduced the number of paired synapses. In animals treated with drug vehicle solution, the number of paired synapses decreased to 15.9 ± 2.0 and 17.0 ± 1.8 in the middle and basal turns respectively (Figure 5B,C). There was also a significant increase in the proportion of orphan synapses in these regions (10% and 8% respectively; Figure 5D).

Treatment with CCG-4986 significantly (*p* = 0.0085) reduced the loss of synapses in the middle turn (Figure 5B) but did not improve the survival of afferent synapses in the basal turn (Figure 5C). In the middle turn, the number of paired synapses improved to 21.1 ± 1.3 synapses per IHC in CCG-4986-treated animals, which was similar to the number of synapses in non-noise exposed controls (Figure 5B).

### 2.6. Spiral Ganglion Neuron Counts

**Figure 6:**
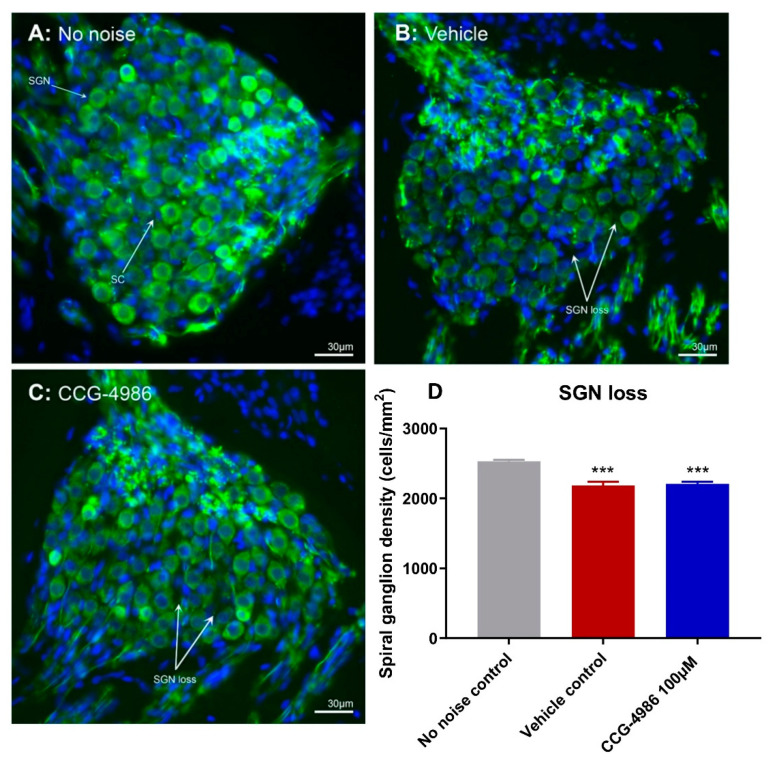
Loss of spiral ganglion neurons (SGN) after noise exposure. (**A**–**C**) Representative images of Rosenthal’s canal in the middle turn of the cochlea. (**A**) Control non-exposed animals, (**B**) Noise-exposed vehicle-treated, (**C**) Noise exposed CCG-4986 treated. SGN were immunostained with the neurofilament antibody (cytoplasm, green) and Hoescht (nucleus, blue). White arrows point at spaces with missing SGN. Abbreviations: SC, satellite cell; SGN, Spiral ganglion neuron. (**D**) Average SGN densities in the middle turn of the cochlea in noise-exposed vs. non-exposed animals. Data presented as mean ± SEM. No noise control, *n* = 10; Vehicle control, *n* = 16; CCG-4986, *n* = 17. *** *p* < 0.001; One-way ANOVA followed by Holm–Sidak post hoc test.

At ambient sound levels (non-exposed controls), the average SGN density in the middle turn of the cochlea was 2530 ± 72 cells/mm^2^ (Figure 6A,D). Noise exposure induced a significant (*p* < 0.0001) loss of SGNs in both vehicle- and drug-treated animals (Figure 6B–D). CCG-4986 treatment did not reduce the loss of SGN, and the average cell densities for CCG-4986 treated (2209 ± 97 cells/mm^2^) and vehicle controls (2183 ± 161 cells/mm^2^) were similar (Figure 6D).

### 2.7. Auditory Brainstem Responses and the Effect of Treatment 24 Hours after Noise Exposure

**Figure 7:**
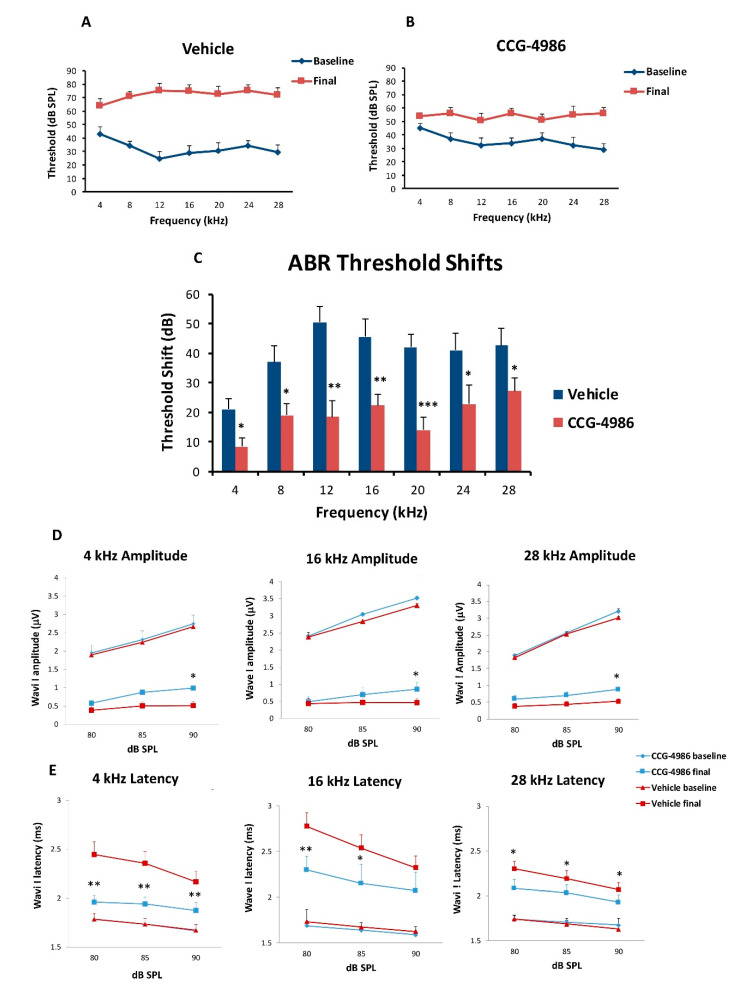
Baseline and final ABR thresholds for 4–28 kHz tone pips in animals exposed to octave band noise (8–16 kHz, 110 dB SPL) for 2 h and non-exposed animals. (**A**) Noise-exposed vehicle-treated animals, (**B**) Animals treated with CCG-4986 (100 μM) 24 h post-exposure. (**C**) Comparison of permanent threshold shifts 15 days after exposure in drug- and vehicle-treated animals. Average baseline and final ABR wave I amplitudes (**D**) and latencies (**E**) at suprathreshold intensities in noise-exposed animals treated with CCG-4986 (100 μM) or drug vehicle solution 24 h after noise exposure. Data presented as mean ± SEM. Vehicle control, *n* = 10; CCG-4986, *n* = 9. * *p* < 0.05 ** *p* < 0.01; *** *p* < 0.001. Two-way ANOVA followed by Holm–Sidak post-hoc test (ABR thresholds), Multivariate ANOVA followed by planned contrast comparisons (ABR suprathreshold responses).

We have also investigated the effect of earlier CCG-4986 treatment (24 h after noise exposure) on ABR thresholds and suprathreshold responses. Like the previous study, exposure to octave band noise (8–16 kHz) for 2 h resulted in 40–50 dB PTS in vehicle-treated (control) animals at frequencies above 4 kHz (Figure 7A,C). However, the administration of a small molecule RGS4 inhibitor, CCG-4986 (100 μM), 24 h after noise exposure reduced PTS by up to 32 dB (Figure 7B,C). The greatest effect was observed at mid-frequencies (12–20 kHz), representing the region of the cochlea most damaged by noise exposure. Average threshold shift was reduced by 32 dB at 12 kHz (*p* = 0.0025), 23 dB at 16 kHz (*p* = 0.01), and 28 dB at 20 kHz (*p* = 0.001). A significant improvement (12–18 dB; *p* < 0.05) was also observed at other test frequencies (Figure 7C).

We have also observed improved suprathreshold responses (ABR Wave 1 amplitudes) after treatment with CCG-4986 24 h post-exposure (Figure 7D,E). The amplitudes and latencies of ABR Wave I were analysed at suprathreshold levels (80–90 dB SPL) in different frequency regions of the cochlea (4 kHz, 16 kHz, and 28 kHz) to assess auditory nerve function. Noise exposure reduced ABR Wave I amplitudes by 60–70% and increased latencies by up to 25% (Figure 7D,E).

CCG-4986 treatment slightly improved Wave I amplitudes compared to vehicle-treated rats in all three test frequencies (90 dB; *p* < 0.05; Figure 7D). In addition, Wave I latencies were reduced after treatment with CCG-4986 (Figure 7E), suggesting partial recovery of neural function.

## 3. Discussion

Our study demonstrates the cochlear rescue effect of CCG-4986 treatment in rats up to 48 h after traumatic noise exposure. This novel treatment is based on enhanced endogenous adenosine A_1_R activity in the cochlea. CCG-4986 is a small molecule RGS4 inhibitor which disrupts the signalling complex (RGS4/Neurabin) that regulates A_1_R activation [18]. We have shown that immunolocalisation of this molecular complex in sensory hair cells and spiral ganglion neurons corresponds to A_1_R distribution in the rat cochlea [19,20]. Local intratympanic administration of CCG-4986 48 h after acoustic overexposure mitigated noise-induced threshold shifts by 10–19 dB, which is considered clinically significant. This otoprotective effect was greater when the CCG-4986 treatment was delivered earlier (24 h after noise exposure), effectively reducing moderate-severe hearing loss to mild hearing loss. CCG-4986 administration also improved suprathreshold responses in noise-exposed animals (increased amplitudes and reduced latencies of ABR wave I), suggesting partial recovery of auditory nerve function. The treatment enhanced the survival of sensory hair cells in the noise-exposed cochlea and mitigated noise-induced loss of afferent synapses in the frequency-specific regions. Noise-induced loss of spiral ganglion neurons was, however, irreversible. The present study thus demonstrates that the RGS4 inhibition is the promising strategy for the treatment of noise-induced cochlear injury and introduces a novel paradigm for the treatment of NIHL and other forms of SNHL based on regulation of GPCR.

### 3.1. Drug Delivery to the Inner Ear

The intratympanic method of drug delivery to the round window membrane has two principle advantages. Firstly, it precludes off-target effects of A_1_R activation in cardiovascular and other tissues, which is an important caveat for systemic administration. Secondly, poloxamer-407 is liquid at low temperatures, but becomes a gel at body temperature which allows slow drug release to the cochlear perilymph [21]. The small size of CCG-4986 (375 g/mol) and its apparent otoprotective effect suggest that this drug readily crosses into cochlear perilymph through the round window membrane. However, further studies are required to establish CCG-4986 concentration in cochlear fluid compartments after intratympanic injection and its pharmacokinetic properties.

### 3.2. ABR Threshold Shifts and Suprathreshold Responses are Mitigated by CCG-4986

CCG-4986 treatment 48 h post-exposure produced a cochlear rescue effect by reducing PTS at all test frequencies above 4 kHz. The PTS reduction of >10 dB is considered clinically significant [22]. As expected, the earlier treatment 24 h post-exposure enhanced the rescue effect of CCG-4986 (up to 32 dB at 12 kHz). The window of opportunity to treat NIHL was thus reminiscent of our previous study using an A_1_R agonist adenosine amine congener [23].

Whilst auditory thresholds are considered a good metric of hair cell function, they are poor indicator of neuronal damage in the cochlea [24]. In this study, the changes in wave I amplitude and latency were used as indicators of afferent neural fibre (ANF) integrity. Noise overstimulation leads to a decrease in suprathreshold Wave 1 amplitudes [25] and prolonged latencies [26] due to reductions in synchronous firing, lower discharge rates, and decreased recruitment of the high threshold ANF [27]. Recent studies postulate that the synaptopathy and the loss of ANF is a primary and mostly irreversible event in noise-induced hearing loss [28]. This loss of cochlear afferents is functionally measured by reduced suprathreshold responses, due to preferential vulnerability of high threshold, low spontaneous rate ANF [29]. The diffuse afferent denervation that cannot be detected by measuring auditory thresholds is thought to contribute to poor performance in complex auditory tasks such as speech discrimination in a noisy environment, and thus has been termed “hidden hearing loss” [30]. In this study, treatment with CCG-4986 24 and 48 h after noise exposure reduced the ABR wave I latencies at all test frequencies, but only a minor improvement of wave I amplitudes was observed, suggesting partial recovery of neural injury.

### 3.3. CCG-4986 Improves the Survival of Sensory Hair Cells

Auditory threshold shifts are largely determined by the integrity of sensory hair cells; hence, the quantitative histological analysis of hair cell survival was carried out in the apical, middle, and basal turns of the noise-exposed cochleae. The hair cell population in the apical segment was virtually unaffected by noise exposure, but a significant loss of OHC was observed in the middle and basal segments of the cochlea, the latter being most severely affected. There was very little IHC loss in the apical and middle turns, but almost one third of IHC was missing in the basal turn of the cochlea.

The increased vulnerability of OHC to noise, particularly in the basal turn, have been well documented in the past. "Inappropriate" loss of high frequency hair cells has also been observed in previous studies where the noise exposures targeted lower frequencies [31]. Basal OHC are particularly vulnerable to acoustic insult, likely due to their lower antioxidant buffering capacity leading to increased susceptibility to oxidative stress [32].

CCG-4986 treatment conferred a significant protection from noise-induced OHC loss in the middle segment and both IHC and OHC loss in the basal segment of the cochlea. Hair cell death is primarily mediated by oxidative stress and calcium overload leading to caspase-dependent cell death pathways [33]. We postulate that the RGS4 inhibition by CCG-4986 enhanced endogenous adenosine A_1_R signalling, which in turn improved antioxidant defences and restored calcium homeostasis [3,18].

### 3.4. CCG-4986 Partly Restores Afferent Synapses but does not Prevent Neuronal Loss

In the absence of noise exposure, the vast majority of IHC synapses contain a pre-synaptic ribbon paired with a post-synaptic terminal from a single ANF, and only around 1% of synapses appear as orphaned pre-synaptic ribbons or post-synaptic terminals [25]. The survival of IHC-auditory nerve synapses is considered a sensitive metric for quantitative analysis of afferent innervation in the cochlea [25].

Noise exposure affected only synaptic ribbons in the mid and high frequency regions, whilst IHC synapses in the low frequency region were mostly intact. Treatment with CCG-4986 yielded a robust neuroprotective effect in the middle turn, to such extent that the average number of functional (paired) synapses in drug-treated animals was not significantly different from control animals exposed to ambient noise.

IHC synaptic loss is an acute event that is usually complete within 24 h after noise exposure. It is generally considered that the loss of afferent synapses is irreversible [25,34,35] but, more recently, post-exposure regeneration of afferent synapses has also been reported after intratympanic administration of neurotrophin-3 [36]. Given that CCG-4986 was administered 48 h after acoustic trauma, it is unclear how it prevents synaptic loss or regenerates afferent synapses. Further studies are required to investigate the timeline of IHC synaptopathic injury in the rat cochlea and establish the underlying mechanism of the rescue effect by CCG-4986.

CCG-4986 treatment 48 h post-insult, however, did not protect against SGN loss. SGN loss usually progresses at a much slower rate than the loss of afferent synapses after acoustic overexposure [25]. Since SGN loss was measured only at one time point (16 days after insult), further studies investigating SGN survival months after acoustic insult are required to fully assess the neuroprotective effect of CCG-4986.

### 3.5. Putative Mechanisms of Otoprotection by CCG-4986

CCG-4986 is a small molecule inhibitor of RGS4 which enhances adenosine A_1_R signalling by disrupting the molecular complex (Neurabin/RGS4) that terminates A_1_R signalling [18]. RGS4 is a GTP-activating protein (GAP) that selectively terminates A_1_R signalling at the level of G protein activation, by accelerating the intrinsic GTPase self-mediated hydrolysis and thus reverting Gα and Gβγ subunits to an inactive state [37]. This effectively terminates the intracellular response following adenosine A_1_R activation.

CCG-4986 blocks RGS4 activity by a dual mode of inhibition, through covalent modification of two different surface-exposed cysteine residues. Firstly, CCG-4986 modifies the Cys132 residue near the binding site of Gα protein and competitively prevents Gα protein binding to RGS4, which moderately reduces the binding affinity of Gα protein to RGS4 [37,38]. The second, more dominant mechanism, involves modification of the Cys148 residue located in an allosteric site that causes a conformational change in RGS4 that prevents Gα from interacting with RGS4, subsequently inhibiting GAP activity and thus extending adenosine A_1_R signalling [37].

Chen et al. [18] demonstrated that the scaffolding protein neurabin is required to facilitate the interaction between RGS4 and A_1_R in the brain. In the absence of A_1_R stimulation, RGS4 is localised to the cytosol. After A_1_R activation, RGS4 is recruited by neurabin to the cell surface, forming the A_1_R/neurabin/RGS4 complex that specifically regulates A_1_R signalling.

Our study shows that CCG-4986 can partially rescue the cochlea from noise-induced injury, most likely by enhancing A_1_R signalling. Noise exposure can induce the release of adenosine into the cochlear fluids and lead to up-regulation of A_1_R expression in cochlear tissues [3,39]. Adenosine A_1_R signalling reduces oxidative stress and lipid peroxidation, likely by boosting endogenous antioxidant defences, including superoxide dismutase and glutathione peroxidase activity [3,40]. A_1_ receptors in the central nervous system are known to exhibit an inhibitory tone by preventing neuronal excitability and synaptic transmission [41]. A_1_R activation thus has a potential to directly counteract the main mechanisms of noise-induced cochlear injury, including oxidative stress, calcium overload, and glutamate excitotoxicity [23,33].

There is a certain advantage of CCG-4986 treatment over adenosine A_1_R agonists such as ADAC and R-PIA, which activate A_1_R with greater selectivity than adenosine. Our previous study [23] demonstrated a biphasic dose-response relationship effect of ADAC after systemic administration, otoprotective at doses 100–200 μg/kg and less effective at higher doses. This "effect inversion" might be due to overstimulation of A_1_R causing their desensitisation and internalisation [42]. In contrast, inhibition of the neurabin/RGS4 complex bolsters the A_1_R signalling without causing a change in A_1_R number or affinity [18].

## 4. Materials and Methods

### 4.1. Animals

For this study, male Wistar rats (6–8 weeks) were sourced from the animal facility at the University of Auckland. Animals were housed in standard cages with ad libitum access to food and water, under controlled conditions (constant humidity and temperature, 12-h light/dark cycle). A minimum of two animals were housed together, up to a maximum of four per cage. Animal welfare was continuously assessed during the study to ensure that animal health was maintained at the highest standard. Noise-exposed animals were randomly assigned into drug treatment or drug vehicle-treated (control) group. All experimental procedures were carried out with the approval of the University of Auckland Animal Ethics Committee (approval # 1631, 8 September 2015), in agreement with the Animal Welfare Act (1999).

### 4.2. Auditory Brainstem Responses

Auditory brainstem responses (ABR) were used to determine auditory thresholds in rats prior to noise exposure (baseline) and 14 days after intratympanic drug or vehicle injection (final). The ABR is an auditory evoked potential that represents the synchronised summation of neuronal activity in response to auditory clicks and tone pip stimuli. ABR recordings were made in a custom-built sound isolating chamber (Shelberg Acoustics, Sydney, Australia), equipped with internal ventilation and a light source. Animals were anesthetised with a mixture of Ketamine (25 mg/kg) and Domitor (0.5 mg/kg) given intraperitoneally. The tympanic membrane was checked for signs of infection, physical trauma, or scarring before ABR recording. Only the left ear was used for assessment of ABR thresholds in each animal. Animals were placed on a thermostatically controlled electric blanket during recordings to maintain body temperature at 37 °C. The Tucker-Davis Technologies (TDT) System 3 and BioSig digital signal processing software (Alachua, FL, USA) were used to generate the auditory stimuli. A multi-field magnetic speaker (MF1, TDT) with 10 cm plastic tubing was used to deliver auditory stimuli into the ear. Three subdermal needle electrodes connected to a Medusa RA4LI headstage amplifier (×20 gain) were used to measure ABR responses. The active electrode was placed at the vertex of the scalp, the reference electrode at the mastoid region of the ear of interest, and the ground electrode at the mastoid region of the contralateral ear. Tone pips (5 ms duration, 2 ms rise/fall, presented at a 21/s rate) were used to elicit ABR responses at intensities between 90 dB SPL to 5 dB SPL, presented in decremental 5 dB steps. Tone pip responses were acquired at an alternating polarity sampling rate of 512 and averaged for each sound intensity. A bandpass filter (300–3000 Hz, 50 Hz notch) was applied to all responses. The ABR wave I was used to determine auditory thresholds defined as the lowest sound intensity level capable of eliciting a reproducible waveform. The cut off amplitude was set at ≥120 nV, as consistently reproducible waveforms were obtained at and above this amplitude. ABR recordings were repeated at sound intensities 10 dB above and 5 dB below the threshold in 5 dB decrements to confirm threshold intensity. In cases where the threshold ceiling was exceeded (above 90 dB SPL), these thresholds were arbitrarily assigned a value of 95 dB SPL. ABR assessments were carried out one day prior to noise exposure (baseline) and 14 days after intratympanic injection (final).

The amplitudes and latencies of wave I were assessed at suprathreshold intensities for selected frequencies (4, 16, and 28 kHz) to investigate the effect of CCG-4986 treatment on noise-induced neuronal injury. Animals with final auditory thresholds of 80 dB SPL or lower were included for input-output functional analysis. The amplitude of wave I (peak to trough) was measured at 90, 85, and 80 dB SPL intensities, and latency was measured as the time taken to reach the peak (including 0.3 ms signal transduction time from the speaker to the ear).

### 4.3. Noise Exposure

Twenty-four hours after baseline ABR measurements, animals were exposed to an octave band noise (8–16 kHz) for 2 h at 110 dB SPL. Acoustic overstimulation was carried out in a custom-built sound-attenuating chamber (Shelburg Acoustics, Sydney, Australia), equipped with internal ventilation, light source, and speakers suspended from the ceiling. Frequency and intensity of sound were adjusted by external controls. The speakers were calibrated using a sound level meter (Precision Sound level Meter Type 2235, Brüel & Kjær; Nærum, Denmark) prior to each noise exposure session, with the average sound pressure intensity measuring 110 ± 1.5 dB SPL across the cage floor. Animals exposed to noise were placed inside a conventional rat cage, positioned with a 30 cm distance underneath the speakers, and allowed to acclimatise for 5 min. Sound intensity was gradually increased over a period of 5 min. Control animals were placed in the sound isolating chamber for two hours to control for relocation stress. Afterwards, animals were returned to the animal housing facility and kept at ambient noise levels (55–65 dB SPL) for the remainder of the experimental timeline.

### 4.4. Intratympanic Injections

RGS4 inhibitor CCG-4986 or drug vehicle solution (control) was delivered by intratympanic injection into the middle ear cavity 24 or 48 h after noise exposure. Drug solution was made by dissolving CCG-4986 (ChemBridge™; San Diego, CA, USA) in 1% DMSO and 0.9% saline with the final 100 μM working dilution for intratympanic injection. Control animals were treated with the drug vehicle solution (1% DMSO in 0.9% saline). Drug and vehicle solutions were mixed with 17% *w/w* poloxamer-407 (Sigma-Aldrich) and placed on ice until fully dissolved. Solutions were then aliquoted and stored at −20 °C for later use. Poloxamer-407 allows for slow drug delivery to the cochlea as the solution becomes a gel at body temperature [21].

Prior to drug treatment, animals were anaesthetised with a mixture of Ketamine (25 mg/kg) and Domitor (0.5 mg/kg) injected intraperitoneally and administered one dose of Temgesic (Buprenorphine; 0.05 mg/kg, subcutaneously) for analgesia. Intratympanic injections were carried out using a Hamilton syringe mounted on a micromanipulator arm. The needle was inserted through the posterior-superior quadrant of the tympanic membrane to deliver injection solution into the vicinity of the round window membrane of the cochlea. A total solution volume of 27 μL was slowly injected into the middle ear. The animal was then returned to a recovery cage and left on its side for 30 min to allow the solution to settle onto the RWM in a gel form. Then the procedure was repeated for the contralateral ear. Animals were then given a subcutaneous dose of Antisedan (1 mg/kg) to reverse the effects of ketamine/domitor anaesthesia.

### 4.5. Cochlear Tissue Preparation for Histology and Immunohistochemistry

After the final ABR assessment, animals were euthanized by an anaesthetic overdose (pentobarbitone, 90–100 mg/kg intraperitoneally). The animals were perfused with the flush solution (0.9% NaCl containing 10% NaNO_2_) and then tissue fixative (4% paraformaldehyde (PFA) in 0.1 M phosphate buffer (PB, pH 7.4) overnight at 4 °C. Cochleae were then decalcified using 5% EDTA in 0.1 M PB for 9 days at 4 °C, cryoprotected overnight in 30% sucrose in 0.1 M PB and embedded in optimal cutting temperature compound (OCT). Cochleae were then snap frozen using N-pentane and stored at −80 °C for further processing.

### 4.6. Hair Cell and Ribbon Synapse Counting

The extracted cochleae were decapsulated and micro-dissected into the apical, middle, and basal segments (turns), after removal of the lateral wall, Reissner’s membrane and tectorial membrane. Cochlear turns were transferred into a 48 well plate containing 0.1 M PBS (pH 7.4), permeabilised with 1% Triton X-100 and blocked with 10% Normal Goat Serum (NGS) for 2 h at room temperature (RT). Whole mount tissues were then incubated overnight at RT with the following primary antibodies: rabbit polyclonal anti-Myosin-VIIa (Proteus Biosciences, 1:500), mouse anti-C-terminal binding protein 2 (CtBP2; IgG1; BD Biosciences, 1:500) and mouse anti-Glutamate receptor 2 (GluA2; IgG2; Merck Millipore, 1:500) in antibody solution containing 0.1% Triton X-100 in 0.1 M PBS with 5% NGS. The following day, sections were washed three times for 60 min with 0.1 M PBS and then incubated at RT with the following secondary antibodies: goat anti-rabbit (Alexa 568, 1:500; Invitrogen), goat anti-mouse IgG1 (Alexa 647, 1:500; Invitrogen), and goat anti-mouse IgG2 (Alexa 488, 1:500; Invitrogen) in antibody solution containing 0.1% Triton X-100 and 5% NGS in 0.1 M PBS. Whole mounts were then washed for 60 min with 0.1 M PBS and then mounted on glass slides using Citifluor AFI mounting medium, cover slipped, sealed with nail polish, and stored in the dark at 4 °C. Inner hair cell-auditory nerve synapses were imaged and analysed at three frequency regions (4, 16, and 28 kHz) of the cochlea, based on the distance from the cochlear apex [43]. Immunostained synapses were imaged using confocal microscopy (Olympus FV1000 Live Cell System, Tokyo, Japan) with oil immersion 60× objective (1.35 NA) and 2.6× digital zoom. Images were captured as a z-stack, with the *z* dimension sampled in 0.2 μm steps, imaging frame in the *xy* dimension capturing 10 adjacent inner hair cells. Z-stacks were processed to remove nuclear CtBP2 staining and compiled into a colour composite stack: inner hair cells labelled with Myosin-VIIa (grey), post-synaptic terminals labelled with GluA2 (green), pre-synaptic ribbons labelled with CtBP2 (red). As a max z-projection could potentially obscure juxtaposed paired synapses in the z-dimension, synapses were counted frame by frame in the z-dimension using the cell counter plugin in ImageJ, with markers displayed through the stack to avoid duplicate counts. A paired synapse was defined as a post-synaptic terminal (GluA2) immediately adjacent to a presynaptic ribbon (CtBP2). Any unpaired pre-synaptic ribbons or post-synaptic terminals were defined as orphan synapses. The total number of synapses were counted for 10 inner hair cells and averaged to get the mean number of synapses per IHC. The number of unpaired (orphan) synapses was expressed as a percentage of the total synapse count per IHC.

For hair cell counting, each cochlea was divided into three segments covering the entire length of the cochlea and representing different frequency regions. The apical segment occupied approximately 0–30% from the apex, middle segment 30–75% from the apex, and basal segment 75–100% from the apex. The organ of Corti was imaged with a Zeiss Axioplan 2 epifluorescence microscope (Carl Zeiss, Jena, Germany) with 20× objective (0.6 NA), and captured with a Photometrics Prime sCMOS monochrome camera. Inner and outer hair cells were counted in ImageJ using the CellCounter to mark intact and missing hair cells, with the number of missing cells expressed as a percentage of the total number of hair cells counted. For regions with complete OHC loss, but intact IHC, one IHC was approximated to three missing OHC (one in each row). For regions of absolute hair cell loss, an adjacent length of IHC was measured (3–4 cells) to calculate pixel width per IHC. The distance of the lesion was measured and the number of missing IHC and corresponding missing OHC was estimated.

### 4.7. Spiral Ganglion Neuron Counting

Cochleae designated for spiral ganglion neuron counts were cryosectioned at 12 μm, and every second mid-modiolar section was placed into 0.1 M PBS (pH 7.4) in a 24 well plate. Cryosections were washed with 0.1 M PBS, permeabilised with 1% Triton X-100 in 0.1 M PBS and blocked with 10% normal donkey serum (NDS) for 1 h at RT. Cochlear sections were then incubated overnight at 4 °C with goat polyclonal Neurofilament (NF-L) antibody (2 μg/mL; Santa Cruz Biotechnology, Inc., Dallas, TX, USA) in antibody solution containing 0.1% Triton X-100 and 10% NDS in 0.1 M PBS. The next day sections were washed (3 × 10 min) with 0.1 M PBS and incubated with donkey anti-goat secondary antibody (Alexa Fluor 488, 1:600 dilution; Invitrogen) for 2 h at RT. Sections were then washed with 0.1 M PBS (10 min), incubated with Hoechst 33342 nuclear stain (1 μg/mL in 0.1 M PBS, pH 7.4; Thermo Fisher Scientific) for 15 min, then washed with 0.1 M PBS for 10 min. Sections were mounted on a glass slide in Citifluor AF1 mounting medium, covered with a coverslip and stored in the dark at 4 °C. Tissues were imaged with a Zeiss Axioplan 2 epifluorescence microscope (Carl Zeiss, Jena, Germany) with 40× objective. Images of the spiral ganglion neurons located in the middle turn were captured at two different wavelengths (UV and 488) using a Photometrics Prime sCMOS monochrome camera and merged to identify individual spiral ganglion neurons. The spiral ganglion area, represented by the bony edge of Rosenthal’s canal, was selected using the "free-hand selection tool" in ImageJ. Individual neurons with unambiguous round nuclei in the middle turn of the cochlea were counted in each section and then averaged to determine SGN density for each animal as described previously [44].

### 4.8. Characterisation of Neurabin Expression in the Rat Cochlea

To determine mRNA expression of the two neurabin isoforms (Neurabin I and II), four intact rat cochleae were extracted, decapsulated, and placed into separate Eppendorf tubes containing cold lysis buffer (100 mM TRIS-HCl pH 7.5, 500 mM LiCl, 10 mM EDTA pH 8.0, 1% LiDS, 5 mM dithiothreitol (DTT) and RNase inhibitors) pre-chilled to 4 °C. Cochleae were then homogenised using sterile Teflon mini-pestles. Polyadenylated RNA (mRNA) was extracted using the magnetic Dynabeads^®^ (Oligo(dT)25 (5 mg/mL)) mRNA DIRECT kit (Invitrogen). First-strand cDNA synthesis was carried out in a 20-μl reverse transcription (RT) reaction with random hexamers, dNTPs and Superscript III reverse transcriptase (Invitrogen). The complementary DNA was amplified by PCR with rat-specific primers for neurabin isoforms (Table 1) designed using OligoPerfect™ (Invitrogen). Negative control without reverse transcriptase was included in each PCR run. RT-PCR with a 40 cycle profile was performed as follows: 94 °C denaturation (1 min), 60 °C annealing (1.5 min), 72 °C extension (2 min) steps using PTC-100^™^Programmable Thermal Controller (MJ Research Inc., Waltham, MA, USA). PCR amplicons were separated by agarose gel electrophoresis, and visualised using SYBR safe DNA gel stain (Invitrogen). PCR products were purified by PureLink™ PCR Purification Kit (Invitrogen) and the identity of the amplicons confirmed by DNA sequencing (Centre for Genomics & Proteomics, School of Biological Sciences, the University of Auckland, Auckland, New Zealand).

### 4.9. RGS4 and Neurabin Immunohistochemistry

The immunolocalisation of RGS4 and neurabin I isoform which forms molecular complexes with RGS4 was demonstrated in the rat cochleae using immunofluorescence. Briefly, rat cochlear tissues were cryosectioned at 30 μm using a CM3050 S cryostat (Leica, Germany) and mid-modiolar sections were placed in a 24 well plate containing 0.1 M PBS (pH 7.4) and washed twice for 20 min. Sections were then incubated in blocking and permeabilisation solution (10% NGS, 1% Triton X-100 in 0.1 M PBS) for 1 h at RT, followed by incubation with a mouse monoclonal RGS4 antibody (Santa Cruz Biotechnologies, sc-398658; 2 μg/mL) or rabbit polyclonal neurabin I antibody (Santa Cruz Biotechnologies; 2 μg/mL) overnight at 4 °C. Control sections were incubated with the normal mouse or rabbit IgG (2 μg/mL) instead of the primary antibody. The next day, sections were washed with 0.1 M PBS three times (60 min), followed by the incubation with the secondary antibody (Alexa Fluor 488 goat anti-mouse or anti-rabbit IgG, dilution 1:500) for 2 h at RT. After a washout with 0.1 M PBS (3 × 10 min), cryosections were mounted on glass slides using Citifluor AF1 Mounting Medium, covered with a coverslip and sealed with nail polish. Images of mid-modiolar cochlear cryosections were acquired using a confocal microscope (Olympus FluoView FV1000) and processed with FluoView software (version 2.0c, Olympus, Tokyo, Japan).

### 4.10. Data Analysis

The researchers were blinded for all ABR assessments, tissue collections, and histological analyses. Animals were assigned a subject ID by an independent researcher and allocated into the treatment or vehicle control group using a randomly generated number list (https://www.randomizer.org). Aliquoted injection solutions were labelled only by subject ID. All data were tested for normality using the Shapiro–Wilk Test. Auditory thresholds, ribbon synapse and hair cell counts were analysed using a two-way ANOVA with a post-hoc Holm–Sidak test. Spiral ganglion counts were analysed with one-way ANOVA. Suprathreshold data were analysed using multi-level factorial ANOVA with planned orthogonal contrasts to determine differences between groups. Data are presented as mean ± SEM.

## 5. Conclusions

Intratympanic administration of a small molecule RGS4 inhibitor presents a novel therapeutic strategy that precludes systemic side effects associated with systemic administration of adenosine A_1_R agonists, while demonstrating a strong rescue effect against noise-induced cochlear injury. Translational studies are required to determine clinical potential of this treatment for NIHL and other forms of SNHL. Future studies will need to determine pharmacokinetic and pharmacodynamic CCG-4986 profile in the cochlea after intratympanic administration, its effect on adenosine concentrations in cochlear perilymph, metabolism, and toxicity profile, before considering clinical trials.

## Figures and Tables

**Table 1:** Primer sequences and positions for neurabin I and II isoforms.

Primer	Forward (Sense)	Reverse (Antisense)
Neurabin I (NM_053473)	5′-GGAGCCGTTAGAAGATGCTG-3′ position: 1247–1266	5′-CCCATCCTCATCTTTCTCCA-3′ position: 1766–1747
Neurabin II (NM_053474)	5′-GAGTGGAGAGGTTGGAGCTG-3′ position: 1976–1995	5′-GGAGCTCCTTGAACTTGTGC-3′ position: 2536–2517

Expected amplicon length: Neurabin I—520 bp, Neurabin II—561 bp.
